# On the Endemic Gastro-Follicular Enteritis, or “Summer Complaint” of Children as It Prevails in the United States

**Published:** 1848-05

**Authors:** 


### ECLECTIC DEPARTMENT,

### AND SPIRIT OF THE MEDICAL PERIODICAL PRESS.

---

*On the Endemic Gastro-follicular Enteritis, or "Summer Complaint" of children as it prevails in the United States.* By Edward Hallowell, M. D., Fellow of the College of Physicians of Philadelphia, Member of the Academy of Natural Sciences, &c.

In the following article, which we copy from the American Journal of Medical Sciences, are contributed some interesting and valuable observations relating to the pathology of the affection generally known in this country as cholera infantum, or, popularly, the summer complaint. The observations appear to have been carefully made and recorded, and appended to the original article in the American Journal, is the series of cases upon which the deductions of the writer are based. The latter we omit.

By selecting articles for this department of our Journal, we do not, of course, intend to signify our assent to all, or, indeed any of the propositions therein contained. This is sufficiently understood. But having taken occasion to express a sense of the value of the facts contained in the following memoir, lest it should be inferred, thereby, that we concur in all the views of the writer, we would add that we differ from him in the most important of the conclusions which he bases upon his observations. That the isolated muciparous follicles of the stomach and intestines are the anatomical elements of the mucus membrane chiefly involved in this affection seems highly probable, and we believe that the writer is correct in the opinion that this follicular disease is not, primitively, inflammatory. But that gastro-enteritis is invariably developed in the progress of the affection, constituting its second stage, and requiring depletory treatment, we cannot but regard as an error fraught with evil consequences. The term cholera infantum is employed in a loose way to include all cases of disease, attended by vomiting and purging, which occurs among children during the summer season. Cases of true gastro-enteritis, which should be distinguished as such, are thus embraced, not infrequently, under this title. It is, also, doubtless true that gastro-enteritic inflammation occurs in a certain proportion of cases of cholera infantum as a complication. The importance of discriminating between cases in each of these particulars is obvious; but this is very far from conceding that inflammation is a constant element, and that the treatment should have reference chiefly to its existence. Practice based upon this pathological view, must, in our opinion, often be pernicious. The writer deduces the constancy of inflammation from the pathological appearances (which are limited often to the large intestines) found in all the fatal cases. The appearances, however, as submitted in his report, do not appear to justify the deduction. Vascular injection, softening, and even loss of substance are not unequivocal evidences of inflammation. But admiting that they authorize the conclusion that the large intestine is more or less inflamed in fatal cases, it does not follow that inflammation is an essential, or constant element of the second stage of the disease. Vascular injection or redness, it is generally conceded by pathological anatomists, do not afford reliable information as to the inflammatory condition of parts; softening of the mucus tissue is a phenomenon incident to chronic disease, in both children and adults, of long continuance manifestly not involving inflammatory action in all cases; and the *mucus membrane* of the large intestine thus softened, is apt to undergo loss of substance at various points, which are mere erosions from the mechanical action of the contents of the bowel. But without endeavoring to explain the appearances presented in all cases in this way, the admission that more or less inflammation actually exists in most cases which prove fatal, does not involve the admission that inflammation uniformly exists in cases which recover, and, still less, that it furnishes the most important indications of treatment. It appears to us highly probable that when it exists, it is often due to the irritating properties of the matters delivered from the small into the large intestine. We do not purpose in this connection to pursue the subject, and we have not time nor space to express our views at length. We have hastily penned the foregoing remarks, to suggest considerations and reflections, which, if we are correct in our appreciation of them, will lead the reader to question the correctness of the conclusion at which the writer arrives with respect to the inflammatory character of cholera infantum. An analysis of symptoms and comparison of the results of different methods of treatment, taken in connection with the pathological appearances, disprove, in our opinion, that conclusion. —Ed. Buff. Med. Jour.

Cholera Infantum, or the "summer complaint" of children has been considered peculiar to the United Sates. Billard, in his diseases of infants, alludes to its occasional appearance in Paris. In the United States it prevails to a great extent, and the mortality from it is extreme. It occurs in all our large cities, carrying off several thousand children annually ; it commences in the Southern States in May, and in the Middle and Western about the beginning or middle of June, and continues until near October, the greater number of cases being observed in July and August. It is found chiefly in the lanes annd alleys of our large cities among the poorer classes of society, but those in the higher ranks are by no means exempt from its attacks. It is stated by Dr. Condie, that during a period of fifteen years from 1825 to 1839 inclusive, 3352 infants perished of this disease in Philadelphia, being almost ten per cent. of the whole number of infants under five years who died during that period. In St. Louis, Mo., during the years 1841, 1842, and 1843, 238 children died of it. In 1823, 253 died of the same complaint in Baltimore. The average number of deaths annually in Philadelphia is about 200. The disease is confined almost exclusively to children between four and twenty months of age; cases, however, occur as early as the age of two months, and as late as period as three or five years.

*Causes of the Disease.*—Cholera infantum is considered to be dependent for its production upon a heated, confined, and impure atmosphere, acting "directly upon the skin, and indirectly upon the mucous surface, at a period when the latter is already strongly disposed to the disease from the effects of dentition, and from the increased developement and activity of the muciparious follicles which take place at that period." The circumstances of its origin, however, are involved in doubt, and can only be determined by future and more correct observation. The exciting causes are stated by Dr. Dewees to be improprieties in diet and clothing. He observes also, that it is very often aggravated by worms, but such a complication has not come under our notice.

*General Description of the Disease.*—Cholera infantum may be divided into three stages, based upon its anatomical characters. In their description we shall be guided chiefly by the results of our own observations.

*Symptoms of the first Stage.*—This usually commences with diarrhœa, succeeded by vomiting, or with vomiting and purging; these symptoms are soon followed by fever of a remittent type, with evening exacerbations; the pulse is small, quick and frequent, occasionally full, and sometimes tense; the brain is often affected sympathetically; this condition is manifested by a tendency to delirium; the eyes have a fierce and wild expression, and the face is flushed; the stools in this stage vary much in consistence; at times they are thin and watery, but often pasty or mush-like; their color also differs greatly in the course of the day, and from one day to another; in a number of cases they presented the appearance of chopped egg, upon which boiling water had been poured; occasionally they consisted almost entirely of mucus. The period at which the vomiting is oberved varies; it occurs usually on the second, but often as late as the fourth or fifth day; in some instances there is no vomiting throughout the course of the disease; in one case it did not make its appearance until a few days before death; the matter vomited cousisted of the contents of the stomach, which were returned almost immediately after their entrance into it; in infants at the breast the milk was returned in a curdled state, having an acid smell; in one instance it had the appearance of coffee grounds; the vomiting occurred for the most part three or four times a day, and sometimes oftener.

*Temperature of surface.*—The skin was occasionally moist, more frequently dry, warmer upon the head and abdomen; the latter is mostly warmer than the rest of the body, and often decidedly hot; the temperature of the extremeties is natural, or more generally cool; occasionally it is warm; sometimes the lower extremeties are cool while the upper retain their usual heat. The *respiration*, except in those cases complicated with other diseases, as hooping-cough or measles, was free, with the number of respirations in the course of a minute amounting to 20, 24, 28, 29, 30, 33, 36, 40, 44, 48, 53, 55, 56. 60, 64, 66. When over 30 the respiration was more or less interrupted. The *tongue* in this stage was observed to be moist, but was often red at its tip and edges, and coated at its base with a yellowish or brownish yellow fur.

The *countenance* in the early stage, except when the attack was violent, was good, the eyes being bright and animated; occasionally the child would fall into a sleep from which it was easily roused. There was usually a considerable degree of irritability and restlessness, the little sufferer being pacified with difficulty. The sleep was often disturbed. The abdomen was occasionally tense and tumid, and somewhat painful on pressure; the thirst was often intense; it now and then happened, however, that drink was refused.

*Anatomical characters.*—These consist in an undue developement of the follicles both of the stomach and intestines, or of one of those organs without inflammation of the mucous membrane. Children rarely die of cholera in the early stage; opportunities, therefore, seldom occur of observing the morbid appearances. M. Billard, who had ample opportunities for the study of the diseases of children at the Hospital des Enfans Trouves of Paris, states that he had seen isolated follicles and follicular plexuses of the intestinal tube in considerable numbers and developed without being inflamed in twelve infants; three were aged from eight days to three weeks, two aged two months, the remaining seven were from nine months to one year; the symptoms of the cases he has published correspond so closely with those of cholera infantum, that, to use the language of Dr. Horner, it is evident that they had occurred in this country, they would have been named, and in fact are cases of cholera infantum. M. Billard states, that most of these children had arrived at the period of dentition, so that there appeared to be a remarkable coincidence between the appearance of the teeth, and that of the organic developement of the follicular apparatus of the intestines, the follicles performing an active part in the process of digestion by furnishing the surface of these organs with a fluid which in all probability assists in the elaboration of food. Dogs, he observes, and other carniverous animals remarkable for their digestive powers, possess this apparatus in a high degree of developement.

In a lioness which died in this city some years ago, and of which I had the opportunity of making a post-mortem examination, the isolated follicles of the intestines were one-fifth of an inch in diamater.

The follicles are sometimes found to exist in great numbers from the first period of life, but in general they are not very numerously developed, except at the period above mentioned, or at a still more advanced age. (Billard.) Rœderer and Wagler, in their work *De Morbo Mucoso*, in which they describe the symptoms and anatomical characters of a gastro-follicular enteritis that prevailed in Gottingen in 1760 and 1761, give very beautiful and accurate drawings of the mucous follicles in a state of abnormal developement.

The muciparous glands of the mucous membrane are, it is known, of two kinds, the isolated and the agminated. The isolated follicles are found both in the stomach and intestines, but are much more abundant and less uniformly developed in the former than in the latter; the agminated follicles or glands of Peyer occupy the free border of the intestine; these are rarely affected in cholera infantum. The isolated follicles are found less abundantly in the jejunum, than in the other portions of the small intestine. They are usually about the size of a pin's head, and have not inaptly been compared by Dr. Horner to grains of sand sprinkled over the surface of the intestines. They are elevated above the surrounding surface of the membrane, and have each in their center a small grayish point. They consist simply of a duplicature of the mucous membrane, and are readily effaced by passing the handle of the scalpel over them with some force; on cutting them open they are found to contain a small quantity of fluid. These glands receive each, according to Leiberkuhun, Meckel, and Beclard, an artery, a vein, and a nerve. They occupy both the summit and the interstices of the valvulæ conniventes, and are found disseminated over every part of the intestine, both upon the free and attached surface. (See case of Charles Morand, in Billard, *Maladies des Enfans*, and of Marie Boulefray in the work of the same author, entitled "*De la Membrane Muqueuse gastro-intestinale dans l'etat sain, et dans l'etat inflammatore, researches d'anatomie pathologique sur ses divers aspects, sains et morbids.*" Paris, 1825.)

*Treatment*.—We have thought it proper to intercalate with our own, the opinions of older and more experienced members of the profession, more especially those of Dr. Chapman, Prof. of Practice in the University of Pennsylvania. His acute perception of *symptoms*, appears to have impressed him more fully than most others with the importance of depletory measures in the treatment of this disease. *Preventive means*.—In the beginning of June examine the condition of the gums of the child, and if swollen, lance them and send it into the country. A part of the country should be chosen which is elevated, where the air is pure and dry, and where there are no large streams nor stagnant water. Chestnut Hill, in the vicinity of Philadelphia, offers superior advantages in this respect. If it be impossible to remove the child to the country, especially if the apartments of its dwelling be not fully and freely ventillated, let it be carried into the open squares of the city, or taken frequently across the Delaware in the afternoon. The sleeping apartments of children should be large and well aired. They should sleep upon a matrass or blanket folded upon the floor of the room or crib, and the covering should be light; they should be immersed in a cold bath at least once a day, or be freely sponged in cold water; the mortality among the children of Philadelphla is said to have greatly diminished since the introduction of the Schuylkill water; the child should be confined to the mother's breast, care being taken not to overload the stomach, indeed it often happens that the breast is given to the child when it requires only drink; the stomach then becomes distended and vomiting often ensues. Attention during the first year should be paid to the diet of the mother; she should avoid articles of a flatulent or indigestible character. Dr. Parrish recommends as a preventive means, when predisposition to cholera is suspected, the occasional use of nutricious animal fluids; the sucking of small pieces of salt meat, as ham or dried beef, he observes, will sometimes be found productive of advantage. With the same view of giving tone to the stomach, he recommends that aromatics, as ginger tea, be used habitually during the summer, in those cases in which there is strong reason to apprehend the occurrence of cholera. This treatment may possibly be applicable undercer tain circumstances, as in the case recorded by him, but as a general rule we are not disposed to recommend it. Indeed, he himself observes, that it is applicable in all its details only in those in whom there is every reason to apprehend that the only alternative is between almost certain death and the most careful prophylatic treatment.

*Treatment of the first stage.*—We havs seen that the first stage consists chiefly in an abnormal development of the mucous follicles of the stomach and intestines. This, as observed by M. Billard, is not properly speaking, a true inflammation, being rather an intermediate stage between the normal state, and the state of inflammation. The follicles are simply in an excited condition, induced as there is reason to suppose, by the powerful influence of heat combined with malaria, acting upon the nervous system. The object of treatment, therefore, is to subdue this excited condition, and to restore the healthy state of the skin with which, and the mucous lining of the alimentary canal, there is a powerful sympathy. The functions of the liver, it is probable, are more or less impaired, but of this we have no direct evidence. If the child be not weaned, it should be confined to the mother's breast, care being taken to avoid overloading the stomach, thus adding to the irritation which already exists. If weaned, it should be confined to milk and water, rice, barley, gum, or toast water. The infusion of the benne leaf (Sesamum orientale), is also employed in this city, and is a useful remedy. The gums should be carefully examined and lanced if swollen. The most important point of treatment however, is early removal to the pure air of the country. This should be done as soon as the disease has declared itself. Dr. George B. Wood, Prof. of Materia Medica and Pharmacy in the University of Pennsylvania, states, that during the whole period of his practice, (nearly thirty years,) but one case occurred to him, so far as he can recollect, of a fatal termination of this disease, when the child had been sent into the country in the early stage of it. (*Pract. of Med.*, vol. i. p. 673.

Blisters behind the ears have been highly recommended at this period of the disease, and we believe the practice is attended with decided advantage. Dr. Parrish was the first to suggest them. They should be applied immediately. The advantage arising from the early application of blisters behind the ears is confirmed by the late Dr. Eberle, a distinguished practitioner of this city, favorably known by his writings on the management of children. He observes, "during the last seven years, I have treated but few cases in which I have not at once applied blisters behind the ears, and I may confidently affirm, that since I have adopted this practice, I have been much more successful in the management of the disease than formerly. Their good effects arise, no doubt, from the counter-irritation they produce." The warm bath should be administered every night and morning, and the child on coming out be gently rubbed with a piece of soft flannel; should there be much cutaneous sensibility, a portion of salt and mustard may be added to it. With a view of allaying the vomiting, various remedies have been employed. Injections of salt and water have been recommended by Dr. Dewees. Limewater and milk, and the neutral mixture may be used for this purpose. Dr. Chapman states that he has found the irritability of the stomach most effectually allayed by lemonade in doses of a teaspoonful, frequently repeated. It should be made pretty sour. Great advantage may also be derived from the frequent use of Seltzer or soda water. Dr. Parrish was in the habit of recommending it to be put up in ℥ ss phials, the contents of one of which to be given at a draught. The soda powders may also be employed. Chicken water was also directed with the same view. Stimulants, as strong coffee, sulphuric ether, turpentine, &c., are objectionable. Dr. Condie states, that when the irtitability of the stomach has been excessive he has rarely failed in allaying it by the administration of the acetate of lead, in solution according to the following formula. ℞.—Aq. puræ ℥ j; acet. plumb. grs. v; acid acet. impure. ♏ v; sacch. alb. pur. ʒ iij. Sig. A teaspoonful may be given every hour or two until the vomiting be suspended. We have been in the habit of directing the blue mass mixed up with gum arabic, with a view of overcoming the irritable condition of the stomach, and usually with success, or small doses of calomel, (1-12th of a grain every two or three hours, either alone or in combination with a very minute quantity of opium, 1-16th, 1-20th, 1-30th, 1-40th of a grain.) The blue mass may be given as follows: ℞.—Mass. ex hydrarg. grs. iv; g. acaciæ pulv.; sacch. alb., *aa* ℥ ss; aq. ℥ iss. M. Sig. A teaspoonful every hours.*

If the vomiting be obstinate, a Cayenne poultice or small blister may be applied to the epigastrium. Should the disease continue, we next have recourse to small doses of calomel and ipecacuanha in doses from 1-12 to 1-6 of a grain of the former, and from ¼ to ½ a grain of the latter, every two or three hours; advantage may sometimes be derived from substituting a small quantity of Dover's powders, (from ¼ to ½ a grain,) for the ipecacuanha. We should be extremely cautious, however, in the use of opiates in this or indeed in any stage of cholera infantum. Benefit may be also derived from the application of a bread and milk, or mustard poultice over the whole abdomen, or as advised by Dr. Eberle, a poultice composed of two or three tablespoonfuls of powdered black pepper, with a few teaspoonfuls of Cayenne mixed up with a common poultice. Embrocations to the abdomen and lower extremities, with spirits of camphor, may also be used with advantage, Should there be symptoms of acidity, which may be ascertained by testing the stools with litmus paper, a few grains of magnesia may be given, and should the discharges continue so as greatly to debilitate the patient, two or three grains of prepared chalk may be added to the prescription of calomel and ipecacuanha. We cannot, however, protest too strongly against the injudicious use of astringents in this or any other stage of the disease. We once witnessed a case in which cerebral symptoms of an alarming character supervened in consequence of a sudden arrest of the alvine discharges by means of the chalk mixture, and the child's life became a forfeit.

*Second stage.*—The vomiting which, in the commencement, was more or less frequent, now occurs but seldom, while the diarrhœa continues; the stools vary much in appearance, but are more or less bloody and painful : there is also much restlessness, and the child is observed to draw up its limbs at the time of the discharge; the predominating color of the stools is dark green, looking like chopped spinnach; the color, however, is occasionally lighter, but mixed with portions of a darker hue, or with lumps of yellow more or less curdled. They are often of a bright yellow or chrome color, or of a dark brown or chocolate color, caused by the admixture of grumous blood. The appearance of the stools varies much in the course of the day, the change of color probably depending upon the greater or less quantity of bile and acid in the intestines; the abdomen is more or less tumid and painful on pressure; tenderness of the abdomen, with drawing up of the limbs, and bloody

* Kreosote will be found in a large proportion of cases effectual. —Ed. Buff. Jour discharges are the most important signs in this stage of the affection; the temperature of the abdomen is usually elevated, while that of the extremities is cool; the pulse is small and feeble, or it is frequent and tense; occasionally it is intermittent; as the disease advances, the emaciation already observed progresses, the skin about the neck and thighs hanging in folds; the eyes become sunken in the orbit, and each is surrounded by a dark areola; the nose is sharp, and the lips are shrivelled; the feet become œdematous, and the cutaneous sensibility is so much impaired that flies collect upon the face without causing any uneasiness; petechiæ are occasionally observed at this period; the tongue is dry and incrusted and covered with aphthæ, and deglutition is now more or less painful; the child is often observed to thrust its fingers far back into the mouth, from dryness of the fauces; the appetite becomes greatly impaired, and there is almost constant thirst. Dr. Dewees mentions the eruption of a quantity of minute vesicles upon the chest, which he considers a fatal sign. Dr. Condie states that he has known many instances of recovery, even when the eruption has been most extensive and distinct. We have observed it but in a single instance; the eruption, however, was not confined to the chest, but occurred in other parts of the body. Dr. Chapman speaks of the appearance of pink-colored stools as a fatal symptom; this does not correspond with our own observations.

*Anatomical characters of the second stage.*—These consist essentially in inflammation with softening of the mucous membrane and ulceration of the follicles, more especially of the large intestine. The mucous membrane of the stomach in many cases presents its usual appearance and consistence; in others it is more or less injected and softened, the softening extending occasionally to all the coats, resembling the condition described by Cruveilhier, as characteristic of the disease termed by him *maladie gastro-intestinale des enfans*, and by Jæger, Gairdner, and others softening of the stomach.

Rilliet and Barthez in their work on the diseases of children, notice the correspondence between the symptoms of softening of the stomach as described by Jæger, and those of cholera infantum; but an examination of the cases recorded in this paper will show that this condition of the organ is but rarely observed. The lining membrane of the stomach is not unfrequently covered with a layer of whitish opaque mucus easily scraped off with the handle of the scalpel; the mucous follicles both of the stomach and intestines are more or less apparent; the mucous membrane of the small intestine is occasionally softened, and for the most part pale in the greater portion of its extent, contrary to the statements of Dewees and others, who consider the small intestine as being the exclusive seat of the disease. In one case the portion of intestine inflamed (the lower portion of the ileum), presented a brick-dust color interrupted with alternations of a pale yellow, mottled with red in some points; minute vessels were seen freely inosculating with each other; in other portions the inosculations were less distinct, there being a uniform reddish tinge. In another it was of a dull red, or brick-dust color, minutely injected with red vessels, and in several points, especially upon the surface of the valvulæ conniventes, presented a dotted appearance; it occupied a portion of the intestine four inches in extent from the pylorus. In another case, (Case X.,) the duodenum at its upper portion presented a slight shade of pink, with a few minute arborizations, and in several other instances there was a slight degree of inflammation affecting the duodenum at its upper extremity. There was also a slight inflammation of the glands of Peyer in one or two cases, but for the most part they presented nothing remarkable. The small intestines contained a considerable quantity of orange colored mucus. The large intestine was more or less inflamed and softened in almost every instance; the inflammation existed in the form of bands, and presented a dotted arborescent appearance; in one case these bands were longitudinal; they were five or six inches in length, and several lines in breadth; in another case the bands were about two inches in length, having a minutely arboriform appearance, and were of a deeper red than the surrounding membrane; the first was situated one and a half inches from the cœcum, the second six inches from the first, and extended nearly the whole circumference of the gut; it was three inches in length and very minutely injected, but not so much as to destroy the arboriform arrangement. In most of the cases the redness was diffused with occasional ramifications; in one instance the inflammation occupied the whole extent of the colon; it was of a vivid red throughout, and the membrane was much thickened. The inflammation was here also for the most part diffused, or in the form of bands occasionally presenting a ramiform appearance, the minute vessels freely inosculating with each other. From the margin of the follicles minute vessels were seen to radiate to the surrounding membrane, occupying the entire surface of the intestine, showing that the inflammation commenced in the follicles and extended subsequently to the mucous membrane. The follicles were often found to be more or less ulcerated, the ulcerations sometimes extending as far as the muscular coat; the ulcerations were more numerous and penetrated more deeply in the rectum than in other portions of the intestine; it was often completely riddled with them; we have not observed the surrounding membrane to be implicated to any extent; the mucous membrane was more or less softened in the greater number of cases; in one instance it was thickened; the membrane in this case was intensely inflamed. The coats of the intestine were covered with a layer of mucus, sometimes so thick as to diminish considerably its calibre. It ordinarily contained a quantity of grayish colored fæces of the consistence of gruel. The lungs presented nothing remarkable but a slight engorgement posteriorly except in three cases, one of which was complicated with measles, and the remaining two with hooping-cough; in these cases the usual appearance of lobular pneumonia were present. In one case the patient had been attacked with pleurisy in consequence of exposure to the night air; at the autopsy a considerable quantity of pus was found effused in the cavity of the right pleura, and the lung was more or less disorganized. The peritoneum presented its usual healthy color in all the cases observed; the liver was greatly enlarged in but a single instance, contrary to the statements of most authors who affirm this to be uniformly the case, the gall bladder was more or less distended with dark colored bile staining the finger a deep yellow; the mesenteric glands were not enlarged; the spleen and kidneys presented nothing remarkable. In nearly all the cases the veins of the pia mater were more or less distended; the archnoid was pale and moist, except in one case in which there was a slight opacity at the base of the brain; there was more or less effusion in the subarachnoid cellular tissue, for the most part limpid; occasionally it presented a whitish, opalescent or citron colored appearance; the pia mater was more or less injected, but the injection for the most part appears to have been confined to the larger ramifications; it was easily removed by traction from the surface of the brain; the substance of the brain presented its natural appearance except in two cases, in one of which the central, and in the other both the central and the cortical portions were injected; it was softened in four of the cases; there was little or no effusion in the ventricles; in one instance the lateral ventricles appeared to be quite dry as if wiped with a cloth.

*Treatment.*—We have seen that the second stage is characterized chiefly by tenderness of the abdomen on pressure, drawing up of the limbs, and bloody stools, symptoms which depend upon inflammation of the mucous membrane, more especially of the large intestine. The treatment, therefore, in this stage should be antiphlogistic. There can be no doubt, we think, tnat the mortality from cholera infantum has arisen in a great degree from not keeping its inflammatory character sufficiently in view. We have found inflammation of the mucous membrane of the large intestine in every autopsy that we have made, and evidence of its existence in an advanced stage of the disease clearly demonstrated in all the published cases that have come under our observation,

The chief object of the present essay, indeed, is to direct the attention of the profession to the above fact, and to the importance of antiphlogistic treatment, instead of the purgative plan usually pursued and with such fatal results. When the abdomen is distended and painful on pressure, with a tense frequent or full pulse, v. s. should be resorted to. This is occasionally required at the commencement of the first stage, when there is much cerebral determination ; should v. s. not be sufficient to remove the inflammatory condition, or when the state of the pulse does not indicate it, leeches or cups are to be applied to the abdomen ; we have not been in the habit of directing cups in this affection, but from the great benefit which we have seen result from their use in the lobular pneumonia of children, we have no hesitation in recommending their employment. Mothers naturally object to what they conceive to be a harsh remedy, but by persuation they can generally be induced to submit to it, and the great advantage we have uniformly derived from their employment in the last mentioned disease, we think warrants their use in this. The amount of pain is much less than is supposed, and the quantity of blood is more suddenly and effectually extracted than by leeches, besides which there is no risk of subsequent hemorrage. One, two, three, or four cups may be applied to the abdomen, and repeated should pain on pressure or the bloody discharges continue. It should be remembered that the reaction in children is seldom great, and that the most intense inflammation of the mucous membrane may exist, while the temperature of the skin is but little elevated ; or may be quite cool, and the pulse feeble. Stimulants in these cases are too often given, and may destroy the patient ; the true course is to pursue a cautious antiphlogistic treatment, supporting the patient at the same time by the blandest articles of nourishment. It has been advised to apply leeches to the anus, and this may occasionally be done with advantage. Injections of cold water or iced water, as the severity of the case may require, are recommended by Dr. Miller of New York, and we believe with great propriety. The remedy, he observes, though generally advisable, appears to be best adapted to that period of disease when the alimentary canal has been previously well emptied of its acrid and offensive contents. Should there be reason to suppose the existence of such accumulations, the discharges being watery and mixed with indigestible food, with a tumid state of the abdomen, a grain of calomel may be given every hour or two until the bowels are disturbed. We have observed that the small intestines are but little affected in this disease, and we should think, therefore, that no objection could be made to the use of mild laxatives for this purpose, the treatment being the same as in dysentery. Castor oil, the oleaginous mixture in small doses, or the oil of butter, may also be employed. Injections of the liquor plumbi subacetat. dilut. (ʒ j to a gill of water) are also productive of benefit, or the accetate of lead, in the proportion of five grains to a gill of water, for a child of two years. The internal remedies should be small doses of calomel and ipecacuanha as prescribed in the first stage, or the same dose of calomel combined with ¼ to ½ a grain of Dover's powder every three hours. It may often be advantageously associated with acetate of lead to the amount of from one-sixth to one-fourth of a grain, but we believe this remedy better adapted to a more advanced period of the disease. When there is much irritability, and the skin not perternaturally dry, the ext. hyoscyam. may be substituted for the Dover's powders. Emetics we consider decidedly objectionable in every stage of cholera infantum, notwithstanding that they have been recommended by high authority.

It will be observed that inflammation with ramollissement of the mucous membrane of the stomach, exists in a large proportion of cases, and emetics under such cases must be decidedly injurious. With regard to purgatives the same objection applies. *Laxatives* may occasionally be proper when there is reason to suspect the accumulation of irritating matters in the intestine, and of them the best, as above mentioned, is calomel either alone or in combination with a small quantity of Dover's powders (¼ to ½ of a grain,) and followed by a dose of castor oil. We cannot too strongly urge upon the young practitioner the necessity of caution in the use opium in this disease, or indeed in any other in which there is a determination to the head. The warm bath is a useful remedy, unless the child be too debilitated ; he should be immersed in it up to the neck, and cloths wrung out of cold water applied at the same time to the head, in order to lessen the determination to the brain. Should the application of cups or leeches to the abdomen not be sufficient to remove the inflammatory symptoms, and the pulse be feeble or the patient much exhausted, blisters may be resorted to, but great care should be exercised in their employment lest gangrene ensue. We have seen very troublesome consequences arise from the application of blisters to children, and in one instance we think the child lost its life from their imprudent use. They should never be suffered to remain more than three hours, and after their removal, a large emollient poultice should be applied over the whole abdomen. Should there be symptoms of cerebral congestion manifested by stupor, rolling about of the head, or a disposition to coma, cloths wrung out of cold water, or vinegar and water, or a mixture of equal parts of lead water and spirits of wine should be applied to the head. Should these symptoms continue, leeches in small numbers may be applied to the temples or behind the mastoid processes. Counter irritation should also be made by stimulating pediluvia, or the application of sinapisms to the extemities.

Cholera infantum not unfrequently becomes chronic, the symptoms being very much the same as those of ordinary diarrhœa, or the patient may sink into a typhoid state. The remedies in this case consist of the warm bath, to which salt, mustard, brandy or Cayenne pepper may be added, counter irritating applications to the abdomen, and the internal use of mild astringents. When the diarrhœa is such as to exhaust the patient. the cretaceous preparations may be cautiously employed, great care being taken not to arrest the discharges too suddenly.

The following prescription has been proposed by Dr. Parrish for that purpose. ℞.—Potassæ sub-carb, ℈j ; gum acaciæ, ʒ j ; tr. opii, gtt. vj ; aq. cinnam, ℥iss ; sacch, alb., ʒj. M. A teaspoonful to be taken every two or three hours. Should this not be sufficient, the following may be employed· ℞.—Test. ostrear. ppt., ʒ iss; gum acaciæ, ʒ j; tr. thebaic, gtt. x; sacch. alb., ʒ j; aq. pur, vel aq. cinnam., ℥ iv. M. Sig. A teaspoonful every two hours.

Dr. Condie states that he has derived great advantage from the use of charcoal in chronic cases of this affection, when the discharges were of a dark color, acrid and offensive. He employs it in combination with powdered rubarb, ipecacuanha, and the extract of hyoscyamus, according to the following prescription. ℞—Carb. ligni, ʒ j a ʒ ij; pulv. rhei, ℈ ij; ipecac, grs. iv a grs. xij. M. ft. chart. xii. Sig· One to be taken every three or four hours. Lime water and Milk may also be given in these cases, also equal parts of charcoal and magnesia.

Care ought to be taken not to continue the use of the charcoal too long, as serious accidents are said to have arisen from its accumulation in the bowels. The infusion of soda and hickory ashes is also said to be beneficial. The syrup of rhubarb may be given in doses of from twenty drops to a teaspoonful every two or three hours, or the powder according to the following formula. ℞.—Pulv. rhei, grs. xv; pulv. ipecac. grs. v; magnes. calcinat. grs. xx; sacch. alb. ʒ j; tr. opii gtt. x; ol. anis. gtt. v. or gtt. vi; aq. ℥iij. M. A teaspoonful every hour or two (Dr. Chapman.) Columbo root in powder or infusion may also be employed. (Dose from two to three grains.) The infusion of the common logwood (hematoxylon campechianum,) Dr. Chapman states, was much employed by Dr. Physick in this stage of the disease. The dose is a teaspoonful. A decoction of the bark of the pomgranate root (puncia granatum,) or of the flowers, is also considered useful. The best of all these vegetable astringents, according to Dr. Chapman, is a strong infusion of the leaves of the dew (rubus trivilis) or of the blackberry root (rubus villosus.) The dewberry is preferable, from its greater strength. The dose is about a teaspoonful. The alum plant (Heuchera Americana,) a common plant in the neighborhood of Philadelphia, has also been highly recommended as an astringent in this affection. Alum, in the dose of one or of two grains with a small quantity of opium may also be employed, or the acetate of lead in combination with the same remedy. Dr. Eberle states that in the advanced stage of this disease, he has occasionally derived considerable benefit from the use of the tartrate of iron according to the following formula. ℞.—Ferri tartrat. gr. xi; syr. zingiberis, ℥ss; aq. pur. ℥ij. M. From twenty to forty drops to be given to an infant four or five times daily. Dr. Chapman advises the sulphate of iron under the same circumstances. ℞.—Ferri sulphat. grs. ij; acid sulph. gtt. x; sacch. ald. ʒj; aq, ℥j. M. Dose a teaspoonful as often as necessary. Dr. Meigs has written favorably of a plunging tepid bath made of the infusion of white oak bark. *American Medical Recorder*, vol. iii. p. 507. He states that it produced a rapid amendment in one case in which he tried it, and a perceptible improvement in another. When the discharges from the bowels are small in quantity, thin, dark colored and highly offensive, with flatulency, the spirits of turpentine may be given. Turpentine in the acute form should never be employed. It is a "deadly remedy." The following is the formula proposed by Dr. Condie. Prescription. —Mucilag. g. acaciæ, ℥iij ; sacch. alb. ʒvj ; spt. æth. nitros, ʒiij ; spt. terebinth. ʒij ; magnes. calcinat. grs. xiij ; spt. lavand. comp. ʒij. M. Sig. A teaspoonful threetimes a day, or oftener when the child is over two years of age. The juniper oil is also considered an excellent palliative for this purpose. Prescription.—Ol. junip. ʒij ; sulph. ether ℥ss ; tr. opii. gtt. xl. M. Sig. From ten to fifteen drops from three to four times daily. The balsam copaiba may also be employed. The dose is from three to five drops. Dr. Parrish states that he has frequently directed an infusion of bark and cinnamon in lime water in the following proportions. Prescription.—Best bark coarsely powdered, ℥ss ; cinnamon ʒij ; lime water ℥ijj. M, It should be suffered to stand a little while and then decanted ; a desert spoonful may be taken several times a day. With this remedy we have no experierience. The bark jacket is also occasionally employed. Injections of the bark may likewise be given. These remedies should be employed only when the symptoms are of a typhoid character, the vital forces being insufficient to sustain the patient. When the child is greatly exhausted, stimulating frictions to the body with flannels wrung out of hot brandy and water, or whiskey should be used, or if the exhaustion be extreme, it may become necessary to resort to stimulants internally, as wine whey, or a weak solution of carbonate of ammonia. Dr. Eberle states that in these circumstances he has derived great advantage from the tincture of cinnamon in doses of from fifteen to twenty drops in some mucilaginous fluid every four hours. When the discharges are very frequent, attended with great exhaustion, the spiritsof ammoniæ aromaticusin combination with chalk mixture is a useful remedy. Dr. Hartshorne, one of the most eminent practitioners of this city, recommends the use of creosote in the advanced stage of this disease. The following is a formula he employs. Prescription.—Creosote, gtt. j. ; test. ostrear. præparat., ʒij. ; g. acaciæ pulv. ℥ss. ; aq., ℥vj. Dose a teaspoonful every two hours for a child two years of age.

Tannin, we would suppose might also be advantageously employed in the chronic form of the disease when the discharges are abundant. It is said to be less likely to irritate the stomach and bowels that the ordinary astringents. The dose for a child of two years is one-fourth of a grain every three hours, or of the pure tannin one-twenty-fourth of a grain repeated as often.

When there is an aphthous condition of the fauces, Dr. Parrish thinks he has found nothing do so much good as a gargle of lime water and bark. Dr. Griffiths, he observes, in some protracted cases of cholera, was in the habit of prescribing scalded lemonade to the child and with a very happy effect.

Great attention must be paid to the diet. If not weaned, the child should be confined to the breast, with the occasional use of barley water, toast, or gum water. So long as the inflammation is inactive, or when it has subsided or has assumed a chronic form, articles of a more nourishing kind may be allowed, as boiled milk, tapioca, arrow root, or sago, or thin oatmeal gruel, or flour boiled in milk; the flour shonld be put in a rag, and then boiled until it becomes hard, and grated. This, we believe to be a most excellent article of diet. We know a remarkable instance of chronic dysentery in the adult, cured by small doses of calomel and opium, and confinement to this diet when all other means had failed. In the chronic form of the disease when the patient is greatly debilitated, the appetite occasionally becomes craving for certain stimulating articles of food, which it may be right to gratify. Dr. Parrish relates several instances of this kind in his lectures. Dr. Wistar, he observes, used to mention the case of a child that was brought to the parlor while the family were at dinner. It was extremely weak, and seemed to be in the last stage of the disease. It showed a strong disposition for some ham which was on the table. The black skin covering the ham was the part which is seemed particularly to desire. It was gratified, and it did not discontinue sucking the piece until it had deprived it of its nutritious juices. From this time it began to recover, and ultimately got well. Dr. Wistar was so convinced of the importance of the above practice that he used to tempt his little patients with small pieces of ham. Some would eat it, others seemed to have no desire for this kind of food. In the latter case he did not press it upon them. Dr. Parrish states that he often prescribed the essence of ham in these protracted cases of cholera. He directs the juice to be bottled up to prevent it from becoming rancid, and to be used as occasion requires. He also relates an instance of a child under similar circumstances that seemed very anxious to eat some butter which was on the table; this child was also indulged, and it continued to devour the butter, lump after lump, until it had made way with the whole. From this time it was allowed as much butter as it desired, and under this plan it recovered.

The same directions with regard to ventilation apply to this as to the first stage.

*Third stage.—Symptoms.* The symptoms indicative of this stage of the affection are an unusual disposition to drowsiness or stupor, rolling of the head, and chewing motion of the under jaw, succeeded by convulsive movements or rigidity, of one or more extremities followed by paralysis. When the disease has progressed thus far, it may be considered almost, if not entirely beyond hope.

*Anatomical characters.*—These consist essentially, in disorganization of the structure of the brain from softening of its tissue. The softening is sometimes general, but is more often confined either to the cortical substance, or to the central portions of the brain and cerebellum. The softening may exist to such a degree as to cause the brain readily to give way on slight pressure, or its substance may be rendered quite diffluent so as to resemble cream. These effects are the result of long continued irritation; the substance of the brain when cut into, usually presents numerous red spots from effusion of blood. The pia mater is more or less injected, and its veins much distended. There is also effusion of serum in the sub-arachnoid tissue, and to a greater or less amount in the lateral ventricles. This, however, is not always the case, the surface being sometimes quite dry.

*Treatment.* — Notwithstanding the hopeless condition of the patient, it is our duty to make use of such means as afford any, even the slightest prospect of relief. These consist in the application of leeches to the temples and to the mastoid processes, with blisters to the nape of the neck. They should be dressed with mercurial ointment. The treatment in fact is the same as in tubercular meningitis, and we think we have derived more benefit from the use of blisters in that affection, than from any other means. Cloths wrung out of cold water, or of vinegar and water, should be at the same time kept constantly applied to the temples,

*Diagnosis.* — Cholera infantum may be confounded with tubercular meningitis, or dropsy of the brain. From this, it may be distinguished by the frequency of the discharges, whereas, in the former affection the bowels are usually torpid, and by a proper acquaintance with the natural history of the disease. In tubercular meningitis the premonitory symptoms are such as indicate an affection of the brain; it occurs for the most part in delicate scrofulous children. Cholera infantum commences with looseness of the bowels. In tubercular meningitis, the cerebral symptoms predominate in the commencement. The child is restless and irritable. and complains of acute pain in the head, referring it chiefly to the forehead: the pain is intermittent, and is usually accompanied with a peculiar cry, which has been considered by Coindet and others as pathognomonic; the sleep is more or less disturbed, and there is frequent tossing about of the hands; the head is rolled from side to side, and there is more or less moaning and grinding of the teeth; delirium is almost a constant symptom, and the countenance assumes a peculiar characteristic appearance; this is so marked that even the nurses at the Children's hospital of Paris, easily recognize the disease. It is only in the advanced stages, that cholera infantum can be confounded with tubercular meningitis when the patient relapses into a state of drowsiness or stupor; which is a prominent symptom of the advanced stage of hydrocephalus, and is often accompanied or preceded by convulsions. Cholera infantum may be confounded with the *typhoid fever* of children. To this affection it bears a close resemblance; it may be distinguished from it, however, by the absence of gargouilliment, of the numerous lenticular spots which in typhoid fever usually make their appearance from the sixth to the twelfth day, by the agitation and slight delirum at night; the prominence of the spleen, the character of the fever, which is more intense, and continues beyond the ninth day; and the existence of the sibilant rale, all of which are prominent, although not constant symptoms in typhoid fever.*

The resemblance between the two diseases is such that it is often impossible to distinguish them apart. Cholera infantum may also be confounded with softening of the stomach. The similarity between the symptoms of gelatinous softening of the stomach, as described by Jæger, and those of cholera infantum, appears indeed to be striking; the coincidence has been observed by Rilliet and Barthez, who do not describe the latter disease as a distinct affection occurring in Paris. The following are the signs of gelatinous softening of the stomach, as laid down by them in their invaluable work. If a child be taken suddenly with obstinate vomitings which persist, with instatiable thirst, with pain in the abdomen, with abundant diarrhœa; if at the same time it emaciates with rapidity, whilst the gastric symptoms *almost exclusively* predominate, we may then infer a gelatinous softening of the stomach.—(Tom. i. p. 467. Art. *Gastrite et Ramollissment de l'Estomac.*)

*Prognosis.*—The prognosis in cholera infantum may be considered favorable when the pulse becomes slower, when the temperature is restored to the surface, when the vomiting ceases, and the alvine discharges become less frequent, and more natural; an opposite opinion may be formed when the pulse continues feeble; the surface remains cold; the discharges become very frequent, resembling the washings of meat, accompanied with great uneasiness and jactitation, or a disposition to stupor; should there be rigidity and a partial loss of power of the extremeties, the patient may be considered almost if not entirely beyond the reach of art.

(*The Cases are omitted.*)

